# Evaluating medicine prices, availability and affordability in Bangladesh using World Health Organisation and Health Action International methodology

**DOI:** 10.1186/s12913-019-4221-z

**Published:** 2019-06-13

**Authors:** Lombe Kasonde, David Tordrup, Aliya Naheed, Wu Zeng, Shyfuddin Ahmed, Zaheer-Ud-Din Babar

**Affiliations:** 1 Health, Nutrition and Population Global Practice, The World Bank, 1818 H Street, NW, Washington, DC 20433 USA; 2 Division of Pharmacoepidemiology and Clinical Pharmacology, WHO Collaborating Centre for Pharmaceutical Policy and Regulation, University of Utrecht, P.O. Box 80082, 3508 TB Utrecht, The Netherlands; 3 Initiative for Non Communicable Disease, Health systems and Population Studies Division, International Centre for Diarrhoeal Disease Research, Bangladesh (icddr,b), 68, Shaheed Tajuddin Ahmed Sarani, Mohakhali, Dhaka, 1212 Bangladesh; 4 Brandeis University, 415 South Street, Waltham, MA 02453 USA; 5 University of Huddersfield, Queensgate, Huddersfield, HD1 3DH UK

## Abstract

**Background:**

Previous studies have shown limited availability of medicines in health facilities in Bangladesh. While medicines are dispensed for free in public facilities, they are paid out-of-pocket in private pharmacies. Availability, price and affordability are key concerns for access to medicines in Bangladesh.

**Methods:**

The World Health Organization/Health Action International survey methodology was used to determine price, availability and affordability of 61 lowest price generic (LPG) and originator branded medicines in public facilities, private retail pharmacies and private clinics across 6 regions of Bangladesh. Medicines for non-communicable and infectious diseases, and both on and off the national Essential Medicines List were included. Prices were compared internationally using Median Price Ratio (MPR).

**Results:**

Mean LPG (originator brand) availability in the public sector, private retail pharmacies, and private clinics was 37%, 63 (4) percent, and 54 (2) percent, respectively. Medicines for Non-Communicable Diseases (NCD) and essential medicines were significantly less available than infectious disease medicines and non-essential medicines, respectively.

Mean LPG (originator brand) MPR was 0.977 in the public sector, 1.700 (3.698) in private retail pharmacies and 1.740 (3.758) in private clinics. Six medicines were expensive by international standards across all sectors.

The least affordable treatments in both private sectors were bisoprolol (hypertension), metformin (diabetes) and atorvastatin (hypercholesterolemia).

**Conclusion:**

Availability and affordability of NCD medicines are key concerns where the burden of NCD is rising. These findings show improvement from earlier studies, but room for further advances in availability and affordability of NCD medicines in Bangladesh. A small number of medicines are consistently expensive across sectors in Bangladesh, suggesting the need for strategies to address prices for certain medicines.

**Electronic supplementary material:**

The online version of this article (10.1186/s12913-019-4221-z) contains supplementary material, which is available to authorized users.

## Background

Access to health care is a fundamental right recognized by governments throughout the world. However, about one-third of the worlds’ population lacks reliable access to medicines, with populations in the least developed countries of Africa and Asia most affected [1]. Improving access to medicines and vaccines could save around 10 million lives a year, 4 million of which in Africa and Southeast Asia [2].

Bangladesh, a lower-middle economy in South Asia, has been experiencing a demographic and epidemiological transition with rapid urbanization and a gradual increase in life expectancy [3, 4]. This shifting pattern has increased the demand for medicines, especially for chronic conditions such as diabetes and hypertension [5, 6].

A publicly funded health care system provides consultations at low cost and medicines free of charge to patients, however anecdotal and limited published evidence suggests availability of medicines is low [7, 8]. Only around half of physicians employed in public hospitals at district to union sub-center level are satisfied with availability of medicines in their facilities [9], suggesting widespread lack of medicines stocks in public facilities. This leads to many patients purchasing their medicines out-of-pocket [10, 11] Indeed, the majority of private health spending in Bangladesh (93% in 2014) is out-of-pocket [12], and as a proportion of total health expenditure, private out-of-pocket expenditure accounts for a substantial proportion (67% in 2014), up from 60% in 1995 [12].

Low availability of medicines in the public sector in Bangladesh is potentially a major barrier to accessing medicines, particularly if private sector prices are unaffordable. Indeed, availability of a sample of 32 essential medicines in public facilities in Bangladesh was the lowest among a comparison with Brazil, Malawi, Nepal, Pakistan and Sri Lanka in 2005 [13]. Although prices were comparatively low in Bangladesh, this was based on a sample of only 19 medicines and the data is now over a decade old. The Bangladesh Health Facility Survey (2014) [9], which collected availability of 14 essential medicines in public and private facilities, is the most recent systematic attempt at quantifying access to medicines in Bangladesh. This survey found substantial variation in availability of different medicines across different public facility types, with some medicines being available in less than 10% of all facilities (e.g. amitriptyline), and some being available almost exclusively in the private sector (e.g. simvastatin/atorvastatin). However, the survey focused on only 14 medicines, and did not address pricing or affordability in the private sector.

Bangladesh is home to a thriving domestic pharmaceutical manufacturing industry, with a highly concentrated market mostly dominated by a few large companies [14]. Competition along with price controls on essential medicines by the national regulatory authority—the Directorate General of Drug Administration (DGDA)— is expected to keep prices to consumers relatively low, [12, 14–17] yet relatively little systematic or recent evidence is available on the topic.

In this context, the present study was carried out to measure prices, availability, and affordability of a basket of 61 medicines across six regions of Bangladesh, using the World Health Organization/Health Action International (WHO/HAI) methodology [18]. The findings of this study have major implications for funders, policy makers, and government authorities to improve access to medicines, rational medicines pricing, equity and affordability for consumers.

## Methods

### Study overview

The WHO/HAI methodology [18] was used to measure medicine price, availability, and affordability across three sectors in six out of the eight geographic regions of Bangladesh. For each of 61 medicines included, pricing and availability data were collected from retail pharmacies and private clinics on originator brand (sold by the company originally holding the original intellectual property) and lowest price generic (LPG, locally or internationally produced generic version) medicines. Public sector procurement prices were collected centrally and from public facilities, while availability was assessed only at the facility level.

### Sampling plan

Six regions of Bangladesh were surveyed: Dhaka (central), Sylhet (north-east), Chittagong (south-east), Dinajpur (north-west), Khulna (south-west), and Barisal (south). Data were collected from public hospitals, private retail pharmacies, and private clinics. For the public sector, in each region the main tertiary teaching hospital was included, along with a central district or specialized hospital if present. Four additional facilities were randomly selected from a list of all facilities with pharmacies or dispensaries at primary and secondary levels within a three-hour drive from the main hospital.

Retail pharmacies and private clinics were selected randomly within five kilometers of each public facility. A list of pharmacies was obtained from DGDA and updated by visiting each area and adding any missing licensed pharmacies. Backup facilities were selected from the same area if during data collection more than 50% of surveyed medicines were unavailable. A list of private clinics was generated manually by visiting each study area. In some rural areas, no private clinics with a dispensing pharmacy were found, and backup clinics were selected randomly from the main urban center.

In total 135 medicines outlets were included in the sample, including 4 retail pharmacy and 11 private clinic backup facilities. Of the 135 outlets, 40 were public hospitals (7 tertiary, 8 secondary, 25 primary-level), 44 were retail pharmacies, and 51 were private clinics. The outlets were distributed across regions with 29 in Dhaka (nine public facilities, nine retail pharmacies, 11 private clinics), 23 in Khulna (seven”, seven “, nine, 23 in Sylhet (six “, seven “, 10 “), 21 in Barisal (six “, seven “, eight “), 20 in Chittagong (six “, seven “, seven “) and 16 in Dinajpur (six “, five “, five “).

### Selection of medicines

Sixty-one medicines were included in the survey (Additional file 1: Table S1). The WHO/HAI Global Core List, which is a list of 14 medicines selected based on global disease burden and included in all WHO/HAI surveys; and 47 Supplementary List medicines based on local need and disease burden, existing availability and utilization. The Supplementary List was generated during a consultative workshop with experts from the World Bank (author LK); ICDDR,B (authors SA, AN); a WHO/HAI consultant (ZB) and a national advisory group consisting of pharmacists and doctors, academics, officials from the Ministry of Health, and a representative from the Consumers Association of Bangladesh. All 61 medicines are off-patent in major global markets. For the analysis, survey medicines were stratified by use (infectious disease, non-communicable disease and uncategorized medicines not belonging to either category: Diclofenac [NSAID], Ibuprofen [NSAID], Paracetamol [analgesic/antipyretic], and Dextrose in Sodium Chloride [parenteral solution]) and are listed in supplementary data Additional file 1: Table S1.

### Survey tool and data collection

Data collection forms, derived from the International Medicines Price Workbook (Version 6.1), were piloted in 15 facilities (4 public facilities, 8 private retail pharmacies, and 3 private clinics). A training workshop for data collectors was conducted by one of the authors (ZB). Data collection was started in Dhaka, and subsequently collected simultaneously in all regions, managed and overseen by four authors (ZB, LK, AN, SA).

In public hospitals, data collectors assessed availability and recorded brand and manufacturer of generic medicines at the central medicines store. Purchase price of medicines procured directly by hospitals were also collected, along with prices paid to EDCL. Prices to hospitals of medicines procured centrally in private markets were obtained directly from the Central Medical Stores Depot (CMSD).

Price and availability of originator brand and LPG were assessed in retail pharmacies and private clinics. Unit price per tablet, capsule, or vial was derived from pack size found. Pack size was specified in advance, but data collectors were allowed to select a different size if not available. Formulation strength was specified in advance, but if not found the medicine was listed as unavailable. Area supervisors checked all data collection forms on the day of facility visits, and same day repeat visits were undertaken in 20% of facilities. Data entry was done in duplicate by ICDDR,B staff.

### Data analysis

Pricing and availability was calculated on the full sample, including private retail pharmacies, private clinics and public facilities in which less than 50% of the surveyed medicines were available, in accordance with the WHO/HAI methodology. Availability was expressed as the percentage of surveyed facilities stocking each medicine. Mean availability of each medicine was calculated as the number of facilities stocking the medicine divided by the number of facilities within each sector, while mean availability for each sector was defined as the mean of this value for all 61 medicines surveyed.

The Median Price Ratio (MPR) is calculated as the median unit price of an individual medicine across all facilities within a sector (converted to US$), divided by the 2014 supplier International Reference Price (IRP) from the Management Sciences for Health (MSH) Drug Price Indicator Guide [19]. IRPs are the medians of recent procurement or tender prices offered by not-for-profit suppliers to developing countries for multisource products. Medicines in the present survey are off-patent in Bangladesh and consequently the generic IRP was used as reference price for both originator brand and generic products. At least four price observations from different facilities are needed for calculation of the MPR, except for procurement data where one price is sufficient [20, 21]. The price variation across facilities was summarized by the minimum, 25th and 75th percentiles, and maximum prices values relative to the IRP. Generally, an MPR of 1 or less is interpreted as efficient procurement in the public sector, while an MPR below 3 is considered acceptable for the private sector [12, 13]. The exchange rate for MPR calculations was US$1 = Tk 78.75 (Bangladesh taka: Bangladesh official gazette, December 15, 2015).

For both availability and MPR, mean values across sectors and medicine types were compared with F-tests, and regression models were applied to examine factors (sector, region, global or supplementary list, essential or non-essential medicines list, infectious or non-communicable disease [NCD] or uncategorized medicine) affecting availability and prices of medicines. The general regression models were expressed as:

$$ y={\beta}_0+{\beta}_1 Sector+{\beta}_2 Type+{\beta}_3 Global+{\beta}_4 Essential+{\beta}_5 Region+\varepsilon $$

Where y is the dependent variables of availability or MPR, *sector* is the matrix of measure of the sector (1 = public sector, 2 = private retail pharmacies, and 3 = private clinics; the public sector as the reference group), type is the matrix of type of medicine (1 = medicine for infectious disease, 2 = medicine for non-communicable disease, and 3 = uncategorized medicine; medicine for infectious disease as the reference group), global is a dummy variable measuring whether the medicine was on the global core list (0 = supplemental list, 1 = global core list), essential is a dummy measuring whether the medicine was on the essential medicines list or not (0 = not in the essential medicines list, 1 = in the essential medicines list), and region is a matrix of regions with Barisal as a reference group. βs are associated coefficients or coefficient matrixes, and ε is the random noise. In the model for MPR, as all the coefficients for regions were not statistically significant, we reported results without the dummy variables for regions.

### Affordability calculations

Affordability in both private sectors is measured by the number of days the lowest paid government employee must work to purchase standard treatment regimens using the collected price data. Cost of medical treatment and affordability for 10 acute and chronic clinical conditions, as determined by the WHO/HAI methodology, was calculated: Adult respiratory infection, paediatric respiratory infection, asthma, diabetes, depression, hypertension, hypercholesterolemia, arthritis, anxiety, and peptic ulcer. Cost of antibiotics for acute illnesses were calculated for 7 days, while cost of medicines for chronic illnesses were calculated for one month of treatment. The daily wage of the lowest paid government worker in Bangladesh is Tk 275 (US$ 3.50).^1^

## Results

### Availability of medicines

Three medicines were not found in any facilities in any sector (isosorbide dinitrate, nevirapine and simvastatin), while another five medicines were not found in any public facilities (aciclovir, diethylcarbamazine citrate, fluoxetine, metoclopramide, and pyrimethamine with sulfadoxine).

Mean availability of LPG medicines across all facilities in the public sector, private retail pharmacies, and private clinics was 37%, 63%, and 54%, respectively (*p* < 0.01, Table 1). No originator brand medicines were found in public hospitals. Mean availability of originator brand products in the private sector was low (4% in retail pharmacies and 2% in private clinics).

**Table 1:** Overall Mean Percentage Availability of Survey Medicines in Different Sectors and by Different Medicine Types

	Public hospitals (%)	Private retail (%)	Private clinics (%)	*n*
	Lowest price generic			
All	37	63	54	61
MEDICINE type				
NCD	27	57	50	36
Infectious	48	68	56	21
Uncategorized	68	85	83	4
Essential medicines				
In EML	36	62	53	51
Not in EML	41	69	61	10
Sample List				
Global CORE	52	77	71	14
SupplementaRY	33	59	49	47
	Originator brand			
All	n.a.	4	2	61

*Source: Author’s data*

*Note: n.a: not applicable. Originator branded medicines are not found in the public sector. N: number of survey medicines*

Differences were observed by medicine group. In the public sector, NCD medicines were less available (mean 27%) than infectious diseases medicines (mean 48%) or uncategorized medicines (68%). The differences were statistically significant (*p* < 0.01) NCD medicines were also less available in both private sectors but the difference was smaller. Across all sectors, medicines on the national Essential Medicines List (EML) were marginally less available than non-essential medicines (50.3% vs. 57.0%, *p* > 0.05), and medicines on the Global Core List were more available than Supplementary List medicines selected for this study (66.6% vs. 46.9%, *p* < 0.01).

Mean availability was higher in tertiary care public facilities (49%) as compared to secondary (39%) or primary (33%) public facilities (data not shown). Across regions, public sector availability ranged from 30% (Khulna) to 40% (Dinajpur), retail pharmacies from 54% (Barisal) to 73% (Dhaka), and private clinics from 47% (Barisal) to 66% (Dinajpur, data not shown). All differences were not statistically significant (*p* > 0.05).

Regression analysis (Table 2) showed lower public sector availability was significant compared with both private sectors (*p* < 0.001). Across sectors, groups with significantly lower availability were NCD medicines (*p* < 0.001), Supplementary List medicines (*p* < 0.001), and medicines on the EML (*p* = 0.003). Most coefficients for regional variation were small suggesting little overall regional variation, although availability was significantly higher in Dhaka (*p* = 0.002) and Dinajpur (*p* = 0.004) compared with Barisal.

**Table 2:** Regression Model Examining the Effect of Sector and Medicine Type on Availability and Price

	CoeffICIENT	Std. Err.	*t VALUE*	*P VALUE*	95% ConfIDENCE Interval	
			Availability			
PUBLIC SECTOR	Reference					
- PRIVATE RETAIL	0.252	0.025	9.91	< 0.001	0.202	0.302
- PRIVATE CLINICS	0.176	0.026	6.84	< 0.001	0.126	0.227
INFECTIOUS DISEASE	Reference					
- NCD	−0.143	0.023	−6.33	< 0.001	− 0.187	− 0.098
- UNCATEGORIZED	0.145	0.032	4.58	< 0.001	0.083	0.207
- GLOBAL	0.185	0.026	7.26	< 0.001	0.135	0.235
- ESSENTIAL MEDICINES	−0.086	0.032	−2.69	0.007	−0.149	− 0.023
BARISAL	Reference					
- CHITTAGONG	0.052	0.038	1.39	0.165	−0.022	0.126
- DHAKA	0.113	0.036	3.16	0.002	0.043	0.183
- DINAJPUR	0.103	0.037	2.82	0.005	0.031	0.174
- KHULNA	0.030	0.035	0.84	0.404	−0.040	0.099
- SYLHET	0.049	0.037	1.32	0.186	−0.024	0.120
CONSTANT	0.418	0.044	9.49	< 0.001	0.332	0.505
			Median Price Ratio			
Public sector	Reference					
- Private retail	0.768	0.179	4.30	< 0.001	0.415	1.120
- Private Clinics	0.809	0.182	4.44	< 0.001	0.449	1.170
Infectious Disease	Reference					
- NCD	−0.486	0.170	−2.85	0.005	−0.823	−0.149
- uncategorized	−0.507	0.253	−2.00	0.047	−1.010	−0.007
Global	0.659	0.193	3.42	0.001	0.277	1.040
- essential medicines	0.262	0.147	1.79	0.076	−0.028	0.552
- constant	0.889	0.168	5.30	< 0.001	0.557	1.220

*Source: Authors’ Data*

*Notes: STD. ERR. = Standard Error. The number of observations for the analysis of availability was 1098, and for MPR was 144. The models were estimated with robust standard errors*

### Public sector prices

Of the 61 medicines surveyed, 46 were found in one or more procurement order, all non-originator generics. Mean MPR was 0.977 (ranging from 0.131 to 2.672, Table 3).

**Table 3:** Summary statistics on price dispersion relative to International Reference Price across sectors and medicine groups

	Public sector – Lowest Price Generic					
	Source list	*n*=	Mean MPR	Median MPR	Min	Max
Full sample	Global	13	1.208	1.256	0.131	2.362
	Supplementary	33	0.886	0.758	0.147	2.672
	All	46	0.977	0.776	0.131	2.672
NCD drugs only	Global	7	0.955	0.756	0.131	1.940
	Supplementary	19	0.805	0.627	0.147	2.672
	All	26	0.846	0.629	0.131	2.672
Infectious disease drugs only	Global	4	1.697	1.675	1.076	2.362
	Supplementary	12	0.905	0.875	0.271	1.648
	All	16	1.103	1.118	0.271	2.362
Uncategorized drugs	All	4	1.327	1.482	0.784	1.558
Essential Medicines	In EML	39	1.038	0.890	0.131	2.672
	Not in EML	7	0.637	0.631	0.147	1.440
	Private sector retail - Lowest Price Generic					
Full sample	Global	12	2.286	2.330	0.784	4.430
	Supplementary	43	1.537	1.411	0.325	3.890
	All	55	1.700	1.521	0.325	4.430
NCD drugs only	Global	6	2.290	2.465	1.019	3.527
	Supplementary	26	1.301	1.171	0.325	3.338
	All	32	1.486	1.202	0.325	3.527
Infectious disease drugs only	Global	4	2.810	2.983	0.846	4.430
	Supplementary	15	1.950	1.698	0.330	3.890
	All	19	2.131	1.981	0.330	4.430
Uncategorized drugs	All	4	1.370	1.514	0.784	1.668
Essential Medicines	In EML	46	1.777	1.562	0.325	4.430
	Not in EML	9	1.308	1.371	0.379	2.309
	Private sector retail - Originator Brand					
Full sample	Global	2	8.750	8.750	1.391	16.110
	Supplementary	6	2.014	1.400	0.597	5.902
	All	8	3.698	1.489	0.597	16.110
Essential Medicines	In EML	7	1.925	1.391	0.597	5.902
	Not in EML	1	16.110	16.110	16.110	16.110
	Private sector clinics - Lowest Price Generics					
Full sample	Source list	n=	Mean MPR	Median MPR	Min	Max
	Global	13	2.381	2.604	0.784	4.430
	Supplementary	41	1.537	1.470	0.325	3.890
	All	54	1.740	1.573	0.325	4.430
NCD drugs only	Global	7	2.335	2.604	1.019	3.527
	Supplementary	26	1.339	1.067	0.325	3.443
	All	33	1.551	1.213	0.325	3.527
Infectious disease drugs only	Global	4	2.814	2.968	0.889	4.430
	Supplementary	13	1.933	1.698	0.335	3.890
	All	17	2.141	1.981	0.335	4.430
Uncategorized drugs	All	4	1.603	1.531	0.784	2.568
Essential Medicines	In EML	45	1.807	1.624	0.325	4.430
	Not in EML	9	1.405	1.371	0.379	2.568
	Private sector clinics - Originator Brands					
Full sample	Global	2	8.750	8.750	1.391	16.110
	Supplementary	6	2.094	1.545	0.597	5.902
	All	8	3.758	1.634	0.597	16.110
Essential Medicines	In EML	7	1.993	1.391	0.597	5.902
	Not in EML	1	16.110	16.110	16.110	16.110

*Source: Authors’ Data*

*Note: n = Number of survey medicines with MPR values. Mean and median MPR summarize all calculated median price ratios in the indicated groups, with the MPR for each medicine using the median unit price in local currency compared with the International Reference Price in local currency. Minimum and maximum MPR refer to the lowest and highest MPR observed across medicines in each group*

**Fig. 1:**
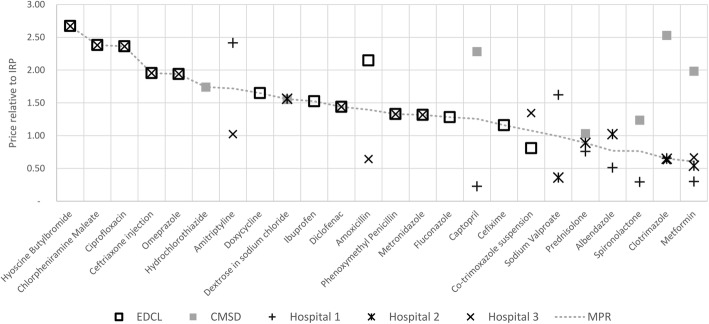
Unit price dispersion relative to International Reference Price in the public sector.Source:Authors’ Data. Note: Ordered by MPR. The data series for individual facilities (except MPR) each show recorded prices divided by the IRP in Taka. MPR is as defined in Methods, i.e. median observed price divided by IRP

Figure 1 shows price statistics for EDCL, CMSD and three public facilities for all medicines in which any price observation was greater than the IRP (24 of 46 medicines). In this sample MPR ranges from 0.60 (metformin) to 2.67 (hyoscine butylbromide). For all other medicines, all observed procurement prices were less than or equal to the IRP. For centrally procured medicines prices were available either from EDCL or CMSD, never both.

Both central (CMSD/EDCL) and directly procured hospital prices were available for 32 medicines (data not shown). Out of these, procurement prices were identical between CMSD/EDCL and hospitals in 15 cases. For most of the remaining medicines, procurement prices were lower in hospitals (13 of 17, of which amoxicillin, captopril, spironolactone, clotrimatzole and metformin are seen in Fig. 1), while EDCL/CMSD prices were lower in a small number of medicines (4 of 17, co-trimoxazole suspension seen in Fig. 1). Up to 10-fold price differences were observed between CMSD/EDCL and directly procuring hospitals.

### Prices in private retail pharmacies

Of the 61 medicines surveyed, 8 originator brands and 55 LPGs were found in four or more private retail pharmacies and were eligible for MPR calculations. Three medicines were excluded on account of being found in fewer than four pharmacies (captopril, metoclopramide, and pyrimethamine with sulfadoxine), and three were not found in any pharmacies (discussed above).

**Fig. 2:**
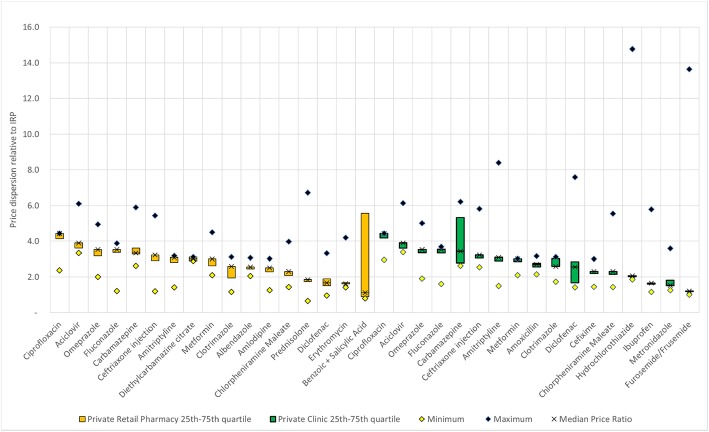
Unit price dispersion relative to International Reference Price for Retail Pharmacies and Private Clinics.Source:Authors’ Data. Note: The price dispersion relative to IRP shows the minimum, maximum, 25th, 50th and 75th quartiles of observed prices in national currency divided by the IRP in national currency. The MPR statistic is represented by X markers. Vertical axis is truncated at 16.0, maximum value of benzoic + salicylic acid in Private Retail Pharmacy is 21.4

A mean MPR of 1.700 was observed across the sample of 55 LPG’s (Table 3). Medicines for which any price observation relative to IRP was higher than 3.0 (17 of 55) are shown in Fig. 2. For the remaining 38 medicines, all pharmacy price observations were less than 3 times the IRP. Substantial variation in unit price relative to IRP is observed for several medicines, most notably benzoic + salicylic Acid where price relative to IRP ranges from 0.8 to 21.4 across pharmacies. A 2–4 fold difference between pharmacies was common (e.g. ciprofloxacin, aciclovir, omeprazole, etc.)

Mean MPR of the eight originator branded medicines was 3.698 (Table 3). Of these, five had higher MPRs than the LPGs (Additional file 5: Figure S2). The largest difference in MPR between the originator product and the LPG was found for diclofenac at + 847% for the originator brand, followed by sodium valproate (+ 80%) and carbamazepine (+ 79%). However, difference in MPR between originator and LPG was not substantial for most medicines. Only two originator brand products, carbamazepine and diclofenac, had MPRs higher than 3.0 (Additional file 4: Figure S1).

### Prices in private clinics

Of the 61 medicines, 8 originator brands and 54 LPGs were found in four or more private clinics. Four medicines were excluded due to nonavailability in at least four private clinics (benzoic with salicylic acid, diethylcarbamazine citrate, fluphenazine decanoate, and pyrimethamine with sulfadoxine), and three medicines were not found in any clinics (discussed above).

Mean MPR across all medicines was 1.740 (Table 3). Medicines with any price observations above three times the IRP are shown in Fig. 2. As in retail pharmacy, many vary 2–4 fold in price across clinics, and a small number exhibit very high variation (hydrochlorotiazide, furosemide/frusemide).

For the eight originator brand medicines, mean MPR was 3.758 (Table 3). Five had higher MPRs than the LPG (Additional file 5: Figure S2). The greatest price difference between originator brand and LPG again was diclofenac at + 513% for the originator brand, followed by sodium valproate (+ 67%) and metronidazole (+ 24%). However, MPR values were mostly consistent between originator brand and LPG for the remaining medicines. Only two originator brands had MPRs greater than 3.0 (carbamazepine and diclofenac sodium).

### Difference in prices across medicine types, sectors, and regions

MPR values were compared across sectors for all medicines available in both sectors. The median) MPR of LPG’s found in both procurement and retail pharmacy (*n* = 45) was 71.0 (96.2) percent higher in retail pharmacy. Similarly, mean (median) MPR of LPG’s in found in both procurement and private clinics (*n* = 44) was 75.7 (104.4) percent higher in private clinics. The median MPR of seven originator brand products was 1.7 (18.2) percent higher in private clinics than in retail pharmacies, whereas no material median difference (2.3/− 0.3%) was observed for 52 LPG’s found in both private clinics and retail pharmacies.

An F-test (Additional file 2: Table S2) confirms mean MPR differences observed across sectors for the whole sample (*p* < 0.01) and for most sub-samples (infectious disease medicines, *p* < 0.01; NCD medicines, *p* < 0.05; on EML, *p* < 0.01; Supplementary List, *p* < 0.05; Global Core List, *p* < 0.05) are significant. Mean MPR for non-essential medicines was empirically lower in the public sector than both private sectors (not significant, note *n* = 10). Mean MPR varied somewhat across regions; however, differences were not significant either within or across sectors (data not shown).

A regression model was specified to determine the effect of these parameters, along with the effect of public versus private sectors (Table 2). The price data was normally distributed. Compared with the public sector, mean MPR values in both private sectors were substantially higher (private retail pharmacy coefficient 0.768, *p* < 001; private clinic coefficient 0.809, *p* < 001). Lower mean MPR of NCD medicines (Table 3) was significant (*p* = 0.003) with a substantial coefficient of − 0.486. Similarly, higher mean MPR for Global Core List medicines was significant (*p* < 0.001) with a coefficient of 0.659. However, the coefficient for medicines on the EML was small and not statistically significant (*p* = 0.197).

MPR values were higher than 3.0 in both retail pharmacies and private clinics for eight medicines (aciclovir, amitriptyline, carbamazepine, ceftriaxone injection, ciprofloxacin, fluconazole, metformin, omeprazole), while one additional medicine (diethylcarbamazine citrate) was above 3.0 in private retail pharmacies only. An MPR greater than 1.0 was found in 18 medicines in the public sector, six of which had MPR values greater than 3.0 in both private sectors (Additional file 3: Table S3).

### Affordability

Total cost of treatment (Table 4) was based on a defined duration of treatment, units per treatment, and the median unit cost of medicines in local currency. The least affordable treatment was bisoprolol for hypertension, followed by metformin for diabetes, and atorvastatin for hypercholesterolemia. The originator brand and LPG were equally affordable for asthma (salbutamol inhaler), while the originator brand was materially less affordable for arthritis (diclofenac) at a total cost of 6–10 times the LPG. Affordability results were comparable across the private retail and private clinic sectors.

**Table 4:** Affordability of 10 treatment regimens based on lowest government wage

Condition	Drug		Treatment parameters		Median unit cost (BDT)		Total treatment cost		Days wages to purchase treatment^a^	
			Duration (days)	Units per treatment	Private Retail	Private Clinics	Private Retail	Private Clinics	Private Retail	Private Clinics
Asthma	Salbutamol inhaler	LPG	as needed	200	0.98	0.98	195	195	0.71	0.71
		Originator			1.15	1.15	230	230	0.84	0.84
Diabetes	Metformin	LPG	30	90	4.00	4.00	360	360	1.31	1.31
Hypertension	Bisoprolol	LPG	30	60	10.00	10.00	600	600	2.18	2.18
Hypertension	Captopril	LPG	30	60	n.a.	2.85	n.a.	171	n.a.	0.62
Hyper-cholesterolaemia	Atorvastatin	LPG	30	30	10.00	10.00	300	300	1.09	1.09
Depression	Amitriptyline	LPG	30	90	1.75	1.75	158	158	0.57	0.57
Adult respiratory infection	Ciprofloxacin	LPG	7	14	15.00	15.00	210	210	0.76	0.76
Paediatric respiratory infection	Co-trimoxazole suspension	LPG	7	70	0.33	0.35	23	25	0.08	0.09
Adult respiratory infection	Amoxicillin	LPG	7	21	6.70	6.63	141	139	0.51	0.51
Adult respiratory infection	Ceftriaxone injection	LPG	7	1	190.00	190.00	190	190	0.69	0.69
Anxiety	Diazepam	LPG	7	7	0.69	0.69	5	5	0.02	0.02
Arthritis	Diclofenac	LPG	30	60	0.88	1.36	53	81	0.19	0.30
		Originator			8.50	8.50	510	510	1.85	1.85
Pain/inflammation	Paracetamol suspension	LPG	3	45	0.33	0.33	15	15	0.05	0.05
Ulcer	Omeprazole	LPG	30	30	5.00	5.00	150	150	0.55	0.55

*Source: Authors’ Data*

*Note: n.a. = not applicable, denotes medicines with fewer than four price observations in the sector. LPG = Lowest Price Generic, Originator = Originator Brand (four or more price observations were only available for Salbutamol inhaler and Diclofenac).* ^*a*^ *Based on average daily wage of Tk 275*

## Discussion

The present study captures availability, price and affordability of 61 medicines across six regions in Bangladesh. Overall, we find availability of surveyed medicines is significantly lower in the public than in both private sectors, and that prices for most medicines in the private sector are not excessive by international standards. A small basket of medicines, however, are consistently expensive across all sectors by international standards. Moreover, when considering unit price and treatment duration, NCD medicines are the most unaffordable medicines. Main points are discussed in more detail below.

### Public sector

We found that on average medicine procurement was efficient in the public sector. The most expensive medicine by international standards was procured at approximately 2.5 times the IRP, while overall less than half of medicines were priced higher than the IRP. Most medicines with high prices relative to International Reference Prices in the private sector were also found to have high prices in the public sector. Consequently, a relatively small sample of medicines, consistent across the three sectors, is associated with higher prices in Bangladesh compared with International Reference Prices.

For most medicines where more than one price was recorded, the variation between EDCL/CMSD and directly procuring hospitals was minimal. However, for certain medicines such as atorvastatin, losartan, metformin and others, substantial variation was observed between EDCL/CMSD on one side and directly procuring hospitals on the other. Local prices relative to International Reference Prices for these drugs were generally not excessive in either sector. Moreover, these medicines generally exhibited high availability in the private retail sector and in the public sector. The lower prices in directly procuring hospitals than in CMSD/EDCL, along with low prices by international standards, suggests certain medicines are being tendered at below-market rates to public hospitals, potentially as part of a commercial strategy to build brand awareness and loyalty for medicines subsequently purchased in the private sector. As most of these medicines are for NCD’s, which patients take over long periods, there is an added incentive for companies to build brand loyalty, particularly as some patients will go on to purchase additional medicines in the private retail sector. A similar situation was described by Azam (2016) who noted that sales to the public sector were less profitable than private sector sales. However companies still market products to the public sector as a strategy to increase prescription share among public physicians, in turn resulting in greater sales when patients purchase these medicines in private outlets [22].

Broadly the findings relating to public sector prices are consistent with studies conducted in neighbouring India, where it was found that medicine procurement systems were efficient and that pooled procurement decreases medicine’ prices [23].

The availability of surveyed medicines in the public sector was overall poor, but higher in tertiary hospitals than in primary-level hospitals. This may have implications for equity of access to medicines, as a smaller proportion of the population will live in the vicinity of tertiary facilities. Availability of medicines in the public sector was substantially lower than in both private sectors.

Contrary to expectation, medicines on the national EML were less available than nonessential medicines in the public sector, and the difference was statistically significant (across all sectors). This is surprising given that EDCL is tasked with producing the majority of essential medicines for supplying public hospitals, and that essential medicines list in general are policy tools for improving access to these medicines. Based on aggregated WHO/HAI survey data from 23 countries, indeed, essential medicines have been shown to be more than twice as frequently available as non-essential medicines [24].

Availability of NCD medicines was substantially less than availability of medicines for infectious diseases in the public sector, and the difference was significant (across all sectors). Among the medicines with highest availability were antibiotics including ciprofloxacin, metronidazole, amoxicillin, doxycycline, and ceftriaxone injection, although some medicines for treating NCDs were also widely available, such as chlorpheniramine maleate, ranitidine, omeprazole, and losartan.

However, this report has demonstrated better availability at low cost of NCD medicines, such as atorvastatin, than previous reports. This suggests public facilities are slowly improving availability of NCD medicines, possibly reflecting increasing demand for NCD care in public facilities and/or establishment of NCD units in primary care facilities. This may be linked with conscious efforts of the Director General of Health Services to strengthen NCD care in Bangladesh [25, 26].

### Private sector

Originator brand products were found in very few outlets. The price range for originator branded medicines was more substantial than for LPG’s, but in most cases the price relative to International Reference Price for the LPG and originator brands were similar or identical. Only in one case was the originator brand more than twice as expensive as the LPG; this notable exception was diclofenac with an approximately eight-fold difference between the LPG and originator brand in both private sectors. Based on these observations it appears originator branded medicines are not a major feature of the pharmaceutical market in Bangladesh, and affordability issues associated with these (sometimes marginally) more expensive medicines are not likely to be substantial.

Availability of LPG medicines in both private sectors was substantially better than in the public sector. There was less difference in availability between NCD and infectious disease medicines in the private sectors than in the public sector. Prices relative to International Reference Prices tended to be higher for infectious disease medicines than for NCD medicines in both private sectors.

A subsample of eight LPG medicines were expensive relative to International Reference Prices in both private sectors. Some of these medicines also exhibit substantial variation in unit price, such as benzoic + salicylic acid in the retail pharmacy sector, and diclofenac in the private clinic sector, warranting further investigation into the causes of variation. In most cases, unit prices for a single medicine would be expected to remain within a narrow range due to the maximum retail price in place in Bangladesh, and the extensive competition in the domestic generics market, which under normal circumstances should lead to pricing near marginal cost.

### Affordability

Only two of the relevant medicines were available as originator brand. For LPGs, it was found that the most expensive treatment regimen (bisoprolol for hypertension) corresponded to more than 2 days of wages in both private sectors. Earlier reports from Bangladesh have examined hydrochlorothiazide for affordability of hypertension treatment, resulting in only 0.1 days’ wages required [13]. The top three most expensive regimens were for NCDs (hypertension, diabetes and hyper-cholesterolaemia), followed by adult respiratory infection treated with ciprofloxacin in both sectors. Cost of medicines to treat adult respiratory infection and all other conditions outside the top three most expensive conditions amounted to less than a day’s wages in both sectors.

The issue of affordability in the private sector is particularly important given the low availability of medicines in the public sector. Affordability can also be severely affected by multiple concurrent illnesses like diabetes and hypertension, and by multiple family members having an illness. According to the present analysis, a co-morbid patient suffering from diabetes, hypertension, and hyper-cholesterolaemia could spend almost 5 days wages per month on medicines, or approximately 25% of gross income for the lowest paid government worker. The level of out-of-pocket expenditure which is considered “catastrophic”, i.e. where households are forced to forego other basic necessities or sell productive assets, is subject to debate, but typically defined as 5–25% of total household expenditure [27]. With this in mind, catastrophic expenditure in Bangladesh may be easily encountered by patients, particularly sufferers of multiple NCD’s. Indeed, in international comparisons, Bangladesh features a high incidence of catastrophic healthcare expenditure [22, 28].

### Limitations of the study

Only medicines with an MSH international reference price could be included in the survey. This is for the sake of comparison. It means that certain medicines and medicines with different strengths could not be included in the study. This has implications for the availability of medicines. It means that the availability data of these medicines may not be meaningful, as these could be available in different strengths.

## Conclusions

In conclusion, we find that availability of medicines in the public sector is generally poor, forcing patients to purchase medicines out-of-pocket in the private sector. With limited options for prepayment and risk pooling, this is likely to lead to catastrophic expenditure and/or under-treatment particularly for lower income households. This highlights the need for increasing insurance coverage of the general population, increasing and improving public service availability or both.

In terms of pricing, a small proportion of medicines were excessively highly priced in the private sector compared with international standards, suggesting a need to explore reasons for this and possible interventions. The government of Bangladesh may for example consider importation of certain medicines. For medicines used to treat NCDs, even though prices were not excessive by international standards, affordability issues were evident due to long treatment duration.

## Additional files

Additional file 1: **Table S1.** List of 61 Core and Supplementary medicines surveyed (DOCX 16 kb)

Additional file 2: **Table S2.** F-Test Values for Comparison of Mean MPR between Sectors (DOCX 13 kb)

Additional file 3: **Table S3.** Medicines with MPR > 3.0 in Private Retail Pharmacies and Private Clinics and with MPR > 1.0 in Public Sector Procurement (DOCX 15 kb)

Additional file 4: **Figure S1.** Comparison MPR of Originator Brand and LPGs in Private Sector Retail Pharmacies (DOCX 25 kb)

Additional file 5: **Figure S2.** Comparison of MPR for Originator Brand and LPGs in Private Clinics (DOCX 25 kb)

## Data Availability

The survey data is available from the lead author of the study.

## Abbreviations

CMSD: Central Medical Stores Depot
DGDA: Directorate General of Drug Administration
EML: Essential medicines list
IRP: International reference price
LPG: Lowest price generic
MPR: Median price ratio
NCD: Non-communicable diseases
WHO/HAI: World Health Organization/Health Action International
