# Supplementary material for: Evaluating medicine prices, availability and affordability in Bangladesh using World Health Organisation and Health Action International methodology
Source: BMC Health Serv Res. 2019 Jun 13;19:383. doi: 10.1186/s12913-019-4221-z (PMC6567665; doi:10.1186/s12913-019-4221-z)
Supplement: Supplementary file 1 — Table S1. List of 61 Core and Supplementary medicines surveyed (DOCX 16 kb) [file 12913_2019_4221_MOESM1_ESM.docx]

SupplementaryTable S1: List of 61 Core and Supplementary medicines surveyed

| **Core medicines surveyed** | | | |
| --- | --- | --- | --- |
| **International non-proprietary name, strength, dosage form** | **Therapeutic class** | **Group** | |
| Amitriptyline 25 mg cap/tab | Antidepressant | NCD | |
| Bisoprolol 5 mg cap/tab | Antihypertensive | NCD | |
| Captopril 25 mg cap/tab | Antihypertensive | NCD | |
| Diazepam 5 mg cap/tab | Anxiolytic | NCD | |
| Metformin 500 mg cap/tab | Antidiabetic | NCD | |
| Omeprazole 20 mg cap/tab | Anti-ulcerant | NCD | |
| Salbutamol inhaler (100 mcg/dose) dose (inhaler) | Anti-asthmatic | NCD | |
| Simvastatin 20 mg cap/tab | Serum-lipid reducing | NCD | |
| Amoxicillin 500 mg cap/tab | Antibacterial | Infectious | |
| Ceftriaxone injection (1 g/vial) vial | Antibacterial | Infectious | |
| Ciprofloxacin 500 mg cap/tab | Antibacterial | Infectious | |
| Co-trimoxazole suspension (8+40 mg/ml) ml | Antibacterial | Infectious | |
| Diclofenac 50 mg cap/tab | Anti-inflammatory | Uncategorized | |
| Paracetamol suspension (24 mg/ml) ml (suspension) | Antihypertensive | Uncategorized | |
| **Supplementary Medicines Surveyed** | | | |
| **International non-proprietary name, strength, dosage form** | **Therapeutic class** | | **Group** |
| Allopurinol 100 mg cap/tab | Cytotoxic and adjuvant medicines | | NCD |
| Aminophylline 100 mg cap/tab | Anti-asthmatic | | NCD |
| Amlodipine 5 mg cap/tab | Antihypertensive | | NCD |
| Aspirin (Acetyl Salicylic Acid) 75 mg cap/tab | Antithrombotic | | NCD |
| Atorvastatin 10 mg cap/tab | Serum-lipid reducing | | NCD |
| Beclometasone (100 mcg/dose) dose | Anti-asthmatic | | NCD |
| Carbamazepine 200 mg cap/tab | Anticonvulsant/Antiepileptic | | NCD |
| Chlorpheniramine Maleate 4mg cap/tab | Antihistamine | | NCD |
| Enalapril 5 mg cap/tab | Antihypertensive | | NCD |
| Fluoxetine 20 mg cap/tab | Antidepressant | | NCD |
| Fluphenazine Decanoate (25 mg/ml) ml (ampoule) | Antipsychotic | | NCD |
| Furosemide/ Frusemide 40 mg cap/tab | Antihypertensive | | NCD |
| Glibenclamide 5 mg cap/tab | Antidiabetic | | NCD |
| Gliclazide 80 mg cap/tab | Antidiabetic | | NCD |
| Glyceryl Trinitrate (nitroglycerin) 0.5 mg cap/tab | Anti-anginal | | NCD |
| Hydrochlorothiazide 25 mg cap/tab | Antihypertensive | | NCD |
| Hydrocortisone 100 mg vial (injection) | Medicine used in anaphylaxis | | NCD |
| Hyoscine Butylbromide 10 mg cap/tab | Antispasmodics | | NCD |
| Insulin neutral soluble (100 IU/ml) ml | Antidiabetic | | NCD |
| Isosorbide Dinitratate 10 mg cap/tab | Anti-anginal | | NCD |
| Losartan 50 mg cap/tab | Antihypertensive | | NCD |
| Metoclopramide 10 mg cap/tab | Anti-emetic | | NCD |
| Nifedipine 20 mg SR cap/tab | Antihypertensive | | NCD |
| Phenytoin 100 mg cap/tab | Anticonvulsant/Antiepileptic | | NCD |
| Prednisolone 5 mg cap/tab | Medicine used in anaphylaxis | | NCD |
| Ranitidine 150 mg cap/tab | Anti-ulcerant | | NCD |
| Sodium Valproate 200 mg cap/tab | Anticonvulsant/Antiepileptic | | NCD |
| Spironolactone 25 mg cap/tab | Antihypertensive | | NCD |
| Aciclovir 200 mg cap/tab | Antiviral | | Infectious |
| Albendazole 400 mg cap/tab | Anthelminthics | | Infectious |
| Amoxicillin suspen (125 mg/5 ml) ml (suspension) | Antibacterial | | Infectious |
| Azithromycin 500 mg cap/tab | Antibacterial | | Infectious |
| Benzoic + Salicylic Acid (6% + 3%) gm (cream) | Antifungal | | Infectious |
| Cefixime 400 mg cap/tab | Antibacterial | | Infectious |
| Chloramphenicol 0.5% ml (eye drop) | Antibacterial | | Infectious |
| Clotrimazole 1% gm (cream) | Antifungal | | Infectious |
| Diethylcarbamazine Citrate 50 mg cap/tab | Antifilarial | | Infectious |
| Doxycycline 100 mg cap/tab | Antibacterial | | Infectious |
| Erythromycin 250 mg cap/tab | Antibacterial | | Infectious |
| Fluconazole 150 mg cap/tab | Antifungal | | Infectious |
| Gentamicin 0.3% ml (eye drop) | Anti-infective | | Infectious |
| Metronidazole 400 mg cap/tab | Antibacterial | | Infectious |
| Nevirapine 200 mg cap/tab | Antiviral | | Infectious |
| Phenoxymethyl Penicillin (pen V.) 250 mg cap/tab | Antibacterial | | Infectious |
| Pyrimethamine with Sulfadoxine (25+500) mg cap/tab | Antimalarial | | Infectious |
| Dextrose in Sodium Chloride (5% + 0.9%) ml (injection) | Parenteral solutions | | Uncategorized |
| Ibuprofen 400 mg cap/tab | Anti-inflammatory | | Uncategorized |

*Source: Authors’ Data*
