# Supplementary material for: Evaluating medicine prices, availability and affordability in Bangladesh using World Health Organisation and Health Action International methodology
Source: BMC Health Serv Res. 2019 Jun 13;19:383. doi: 10.1186/s12913-019-4221-z (PMC6567665; doi:10.1186/s12913-019-4221-z)
Supplement: Supplementary file 2 — Table S2. F-Test Values for Comparison of Mean MPR between Sectors (DOCX 13 kb) [file 12913_2019_4221_MOESM2_ESM.docx]

Supplementary Table S2 F-Test Values for Comparison of Mean MPR between Sectors

| MEDICINE category | Public | Private retail | Private clinics | F-value |
| --- | --- | --- | --- | --- |
| Infectious | 1.103±0.582 | 2.131±1.149 | 2.141±1.168 | 5.71** |
| NCD | 0.868±0.801 | 1.486±0.934 | 1.551±0.978 | 3.02* |
| Uncategorized | 1.327±0.365 | 1.37±0.406 | 1.603±0.740 | 0.31 |
| not In EML | 0.713±0.516 | 1.308±0.731 | 1.405±0.842 | 1.77 |
| EML | 1.093±0.688 | 1.777±1.063 | 1.807±1.078 | 5.47** |
| Supplemental | 0.935±0.659 | 1.537±0.918 | 1.537±0.941 | 4.55* |
| Global | 1.260±0.676 | 2.286±1.208 | 2.381±1.140 | 3.72* |
| Total | 1.028±0.670 | 1.700±1.025 | 1.740±1.046 | 6.90** |

*Source: Authors’ Data*

*Note: Significance level * = 5%, ** = 1%, *** = 0.1%*
