# Supplementary material for: Evaluating medicine prices, availability and affordability in Bangladesh using World Health Organisation and Health Action International methodology
Source: BMC Health Serv Res. 2019 Jun 13;19:383. doi: 10.1186/s12913-019-4221-z (PMC6567665; doi:10.1186/s12913-019-4221-z)
Supplement: Supplementary file 3 — Table S3. Medicines with MPR > 3.0 in Private Retail Pharmacies and Private Clinics and with MPR > 1.0 in Public Sector Procurement (DOCX 15 kb) [file 12913_2019_4221_MOESM3_ESM.docx]

Supplementary Table S3 Medicines with MPR>3.0 in Private Retail Pharmacies and Private Clinics and with MPR>1.0 in Public Sector Procurement

|  | Public Sector Procurement (MPR>1.0) | Private Retail Pharmacies (MPR>3.0) | Private Clinics (MPR>3.0) |
| --- | --- | --- | --- |
| Aciclovir | — | 3.89 | 3.89 |
| Amitriptyline | 1.72 | 3.09 | 3.09 |
| Amoxicillin | 1.40 | n.a. | n.a. |
| Captopril | 1.26 | — | n.a. |
| Carbamazepine | — | 3.34 | 3.44 |
| Cefixime | 1.16 | n.a. | n.a. |
| Ceftriaxone injection | 1.95 | 3.23 | 3.23 |
| Chlorpheniramine Maleate | 2.38 | n.a. | n.a. |
| Ciprofloxacin | 2.36 | 4.43 | 4.43 |
| Co-trimoxazole suspension | 1.08 | n.a. | n.a. |
| Dextrose in sodium chloride | 1.56 | n.a. | n.a. |
| Diclofenac | 1.44 | n.a. | n.a. |
| Diethylcarbamazine citrate | — | 3.01 | — |
| Doxycycline | 1.65 | n.a. | n.a. |
| Fluconazole | 1.28 | 3.53 | 3.53 |
| Hydrochlorothiazide | 1.74 | n.a. | n.a. |
| Hyoscine butylbromide | 2.67 | n.a. | n.a. |
| Ibuprofen | 1.52 | n.a. | n.a. |
| Metformin | n.a. | 3.01 | 3.01 |
| Metronidazole | 1.32 | n.a. | n.a. |
| Omeprazole | 1.94 | 3.53 | 3.53 |
| Phenoxymethyl penicillin | 1.33 | n.a. | n.a. |

*Source: Authors’ Data*

*Note: — = not available; no MPR could be calculated for this medicine/sector due to low availability. n.a. = not applicable, denotes medicines with MPR values less than 1.0 for public sector and 3.0 for both private sectors. All medicines not listed have MPR values of 1.0 or less (public procurement) or 3.0 or less (private retail pharmacies and private clinics).*
