# Supplementary material for: Evaluating medicine prices, availability and affordability in Bangladesh using World Health Organisation and Health Action International methodology
Source: BMC Health Serv Res. 2019 Jun 13;19:383. doi: 10.1186/s12913-019-4221-z (PMC6567665; doi:10.1186/s12913-019-4221-z)
Supplement: Supplementary file 5 — Figure S2. Comparison of MPR for Originator Brand and LPGs in Private Clinics (DOCX 25 kb) [file 12913_2019_4221_MOESM5_ESM.docx]

Supplementary Figure S2 Comparison of MPR for Originator Brand and LPGs in Private Clinics

*Source: Authors’ Data*
